# From molecular mechanisms of glycosphingolipid function to bio-therapeutic applications

**DOI:** 10.1007/s00018-026-06186-1

**Published:** 2026-03-29

**Authors:** Abhijit Saha, Ludger Johannes

**Affiliations:** 1 Center for Cooperative Research in Biosciences (CIC bioGUNE), Basque Research and Technology Alliance (BRTA), Biscay Technology Park, Building 801A, 48160 Derio, Spain; 2 Chemical Biology of Cancer Unit, Institut Curie, Université PSL, U1339 INSERM, UMR3666 CNRS, Paris, France; 3 SAIRPICO Project Team, Inria Center at University of Rennes, U1339 INSERM, Institut Curie, UMR3666 CNRS, PSL Research University, Paris, France

**Keywords:** Glycosphingolipids, Clathrin-independent endocytosis, Glycolipid-lectin (GL-Lect) driven endocytosis, Endocytic trafficking, Retrograde transport, Bio-therapeutic applications

## Abstract

Glycosphingolipids (GSLs) are regulators of membrane organization and trafficking. According to the glycolipid-lectin (GL-Lect) driven endocytosis model, GSLs are part of a mechanism in which they are used by oligomeric lectins from pathogens or cells to build tubular endocytic pits from which clathrin-independent endocytic carriers detach by friction-driven scission. This review revisits the unique architectural logic of GSLs and explores how this underpins mechanisms for endocytic trafficking and signaling. We examine notably how GSLs orchestrate retrograde trafficking from the plasma membrane to the Golgi apparatus and subsequent polarized secretion in specialized contexts such as migrating cells, epithelial tissues, and immune synapses. Despite their importance, GSL functions have remained difficult to explore, because of the scarcity of minimally perturbing, chemically defined tools. We survey recent development in GSL probe design and discuss principles for next-generation tools that retain native function while enabling mechanistic dissection. Finally, we discuss how these advances are reshaping therapeutic strategies, with implications for vaccine delivery, cancer targeting, and lysosomal storage disorders. Collectively, this review reframes GSLs as substrates of membrane organization, with vast untapped potential in both cellular membrane biology and translational medicine.

## Introduction

Glycosphingolipids (GSLs) are among the most abundant lipids in the plasma membrane across diverse life forms [1–3]. A dysregulation of their expression triggers profound developmental and physiological defects [4]. Embedded via their ceramide anchors and diversified at the acyl chains level and through complex glycans, GSLs organize lipid rafts, orchestrate signaling complexes, and shape membrane dynamics [5]. Despite their abundance, GSL biology has remained underexplored for many decades, largely due to structural complexity and lack of minimally perturbing molecular tools. This is changing. Advances in cutting edge chemical biology tools applied to glycobiology are revealing principles that govern their spatial–temporal distribution and functional specificity.

One emerging facet in GSL biology is the glycolipid-lectin (GL-Lect) hypothesis, which proposes that interactions of oligomeric lectins with glycans on proteins and GSLs drive processes of clathrin-independent endocytosis [6]. According to this model, GSLs direct endocytic trafficking and the ensuing polarized intracellular distribution of glycoproteins that contribute to fundamental cellular processes spanning development, host–pathogen interactions, and tumor biology. As new chemical probes and engineered tools unlock the ability to track and manipulate GSLs in living systems, their therapeutic potential is coming into focus — from vaccine delivery for immunotherapy to cancer targeting.

In this review, we begin by unpacking the structural principles and biosynthetic regulation of GSLs, highlighting their distinctive combination of ceramide anchor and glycan diversity. We then explore the landscape of clathrin-independent endocytosis (CIE), with a particular focus on the GL-Lect mechanism. Building on this, we examine how this process influences retrograde transport and polarized secretion in systems such as migrating cells, epithelial barriers, and immune synapses. Then we discuss recent breakthroughs in probe design and perspectives for developing next-generation tools. Finally, we consider how these insights are opening new therapeutic frontiers from vaccine delivery to cancer targeting and lysosomal storage disorders. Altogether, we aim to position GSLs as central elements of membrane biology and biomedical innovation.

## GSLs

**Fig. 1:**
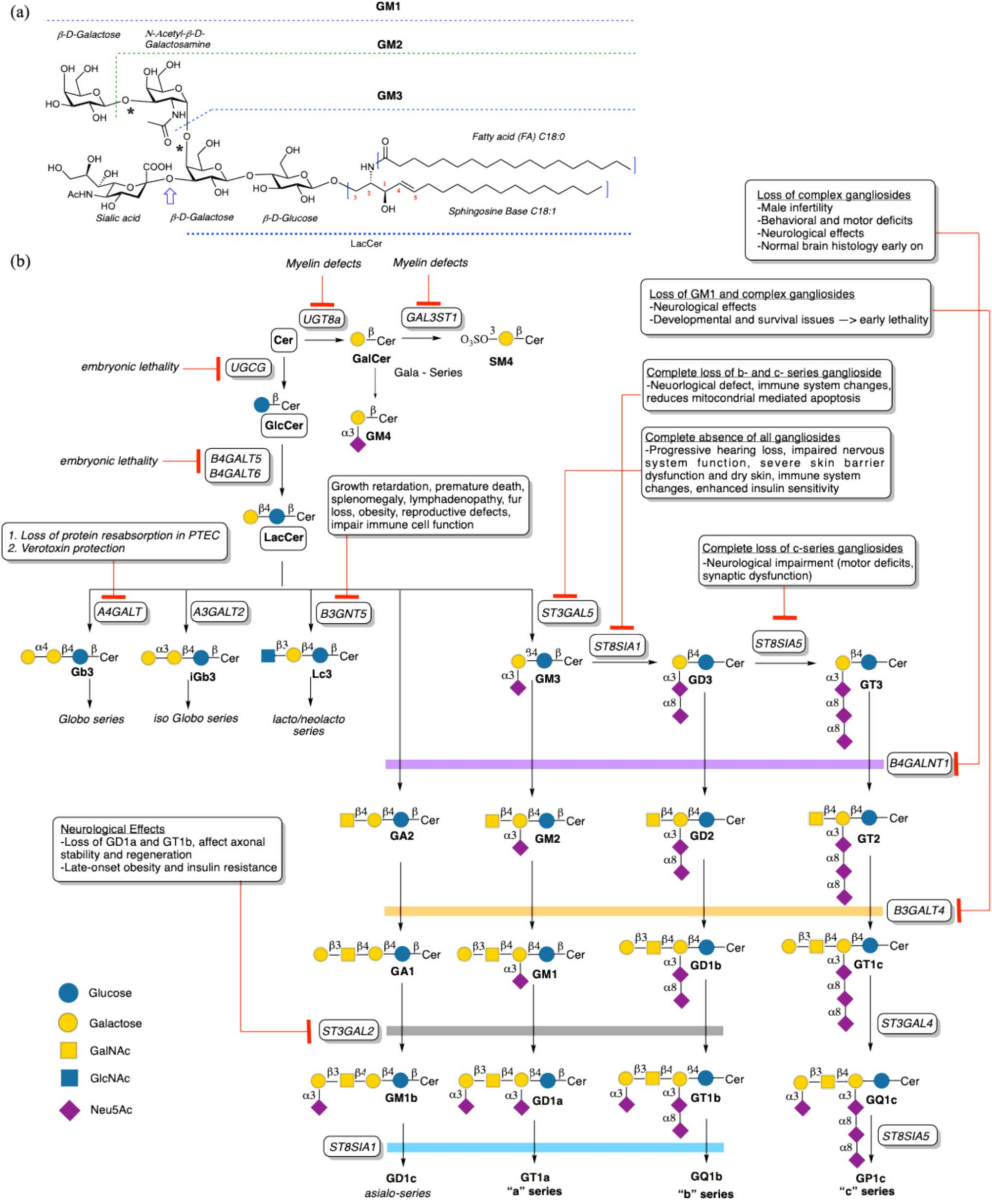
GSL structures, pathways of biosynthesis, and associated mutant phenotypes. **a** Schematic drawing of lactosylceramide, a common precursor of gangliosides in vertebrates*.* GSLs containing one or more sialic acid residues in the glycan moiety are referred to as gangliosides. Sialylation can be achieved on the α3 position (see arrow) and by extension to more complex gangliosides on the β4 position (see star). These extensions result in the formation of complex gangliosides GM1 to GM3. **b** Schematic representation of the GSL biosynthesis tree adapted from Refs. [6, 44]. GSL biosynthesis illustrated from ceramide through sequential glycosyltransferase reactions, highlighting the globo-, isoglobo-, lacto/neolacto-, and ganglio-series. The human brain contains 10–30 times more gangliosides than any other tissue, making them critical for neuronal development, synaptic transmission, and plasticity [45]. Key enzymes (e.g., UGCG, B4GALT5, ST3GAL5, ST8SIA1) and their knockout phenotypes in mice are summarized. For a description of the complete pathways, see Ref. [1]. The figure was prepared using Chemdraw

GSLs are a structurally diverse, amphipathic, and biologically essential class of glycoconjugates, composed of complex carbohydrate moieties that are linked via the* β*-glycosidic bond to a ceramide backbone (Fig. 1a) [1]. The hydrophobic ceramide is embedded within biological membranes, while the hydrophilic glycan headgroups extend into the extracellular environment, enabling participation in cell–cell recognition, signaling, and interactions with pathogens or cellular lectins [1]. GSLs have been recognized as ubiquitous across evolution, with essential roles demonstrated in multicellular organisms such as *Caenorhabditis elegans*, [7] *Drosophila melanogaster*, [8] and vertebrates [9]. Genetic or pharmacological disruption of GSL biosynthesis in these systems results in severe developmental defects or lethality [7–9]. The establishment of interdisciplinary glycobiology approaches combining advanced technologies such as synthetic chemistry [10–12], mass spectrometry [13], and glycan profiling [14], have significantly advanced our understanding of molecular aspects of GSL function.

### GSLs metabolism

Databases such as LipidMaps predict over 3,000 unique GSLs species, documenting the vast structural diversity of this lipid class [15]. GSLs are made in the endomembrane domain. Their biosynthesis starts with the ceramide and then involves the sequential addition of individual sugar molecules transferred through glycosyltransferases [1, 16]. Ceramide biosynthesis occurs through three main pathways: de novo synthesis, salvage, and sphingomyelin hydrolysis. The de novo pathway is the primary route, which starts on the cytosolic leaflet of the endoplasmic reticulum, involving the condensation of fatty acids with sphingosine to create ceramide. Mammalian ceramide synthases 1 to 6 catalyze this reaction. Importantly, each of these six differs in acyl chain length preference [16]. In the salvage pathway, complex sphingolipids are broken down, releasing sphingosine. This sphingosine is then re-acylated by the same ceramide synthases to regenerate ceramide for further GSL production. The sphingomyelin hydrolysis pathway involves the breakdown of sphingomyelin by sphingomyelinases to release ceramide. This occurs at the plasma membrane and may contribute mostly to signaling rather than GSL synthesis.

Once the ceramide is ready, glycosylation is initiated at distinct subcellular sites by the transfer of a single sugars, i.e., glucose, galactose, or fucose in mammalian cells, yielding glucosylceramide (GlcCer), galactosylceramide (GalCer), or fucosylceramide, respectively. These initial glycosylation events generate key precursors for all GSL subclasses, the vast majority being based on GlcCer [1, 17].

Ceramide is transported to the Golgi apparatus via two main routes [18]: either by the ceramide transfer protein (CERT) to the trans-Golgi network (TGN), where it is primarily used for sphingomyelin synthesis, or through vesicular transport to the cis-Golgi [19], where it is glucosylated on the cytosolic face of early Golgi membranes to form GlcCer [18, 20]. Subsequent steps of glycosylation are catalyzed on the luminal leaflet of the Golgi [21]. For this, the initial GlcCer must be flipped from the cytosolic to the exoplasmic leaflet. This occurs via FAPP2, a lipid transfer protein that shuttles GlcCer from the cis-Golgi to the TGN [22, 23]. How FAPP2 “inserts” GlcCer into the exoplasmic leaflet of the TGN remains to be determined. Glycosyltransferases then sequentially elongate the glycan chains, by transferring activated sugar units (e.g., UDP-Glc, UDP-Gal, CMP-sialic acid), giving rise to the diverse array of complex GSLs found on the cell surface (Fig. 1b).

Specifically, GlcCer in the luminal leaflet of Golgi/TGN membranes is galactosylated to produce lactosylceramide (LacCer; Galβ1-4Glcβ1Cer) by β4-galactosyltransferases V and VI [24–26]. LacCer cannot be translocated back to the cytosolic leaflet [27]. Instead, LacCer is the metabolic branch point for the formation of the different classes of complex GSLs (Fig. 1b) [28]. In fact, LacCer is the precursor of GSLs like GA2, GM3, Gb3 and Lc3 which further extended for the synthesis of asialo, ganglio, globo and lacto series, respectively.

To produce GalCer, Cer is galactosylated within the ER lumen [29, 30]. GalCer is then transported to the Golgi complex where it can be sialylated to produce GM4 ganglioside or sulphated to produce sulfogalactolipids [1].

Once synthesized, GSLs embark to the plasma membrane within vesicular carriers [31]. At the cell surface, GSLs are not static: they can be locally remodeled by specific glycosidases, fine-tuning their glycan architecture in response to environmental cues [32]. A fraction of surface GSLs is continuously internalized through endocytic pathways, ultimately reaching lysosomes for turnover. Here, a coordinated suite of glycohydrolases and accessory proteins dismantles the glycan chains step-by-step, releasing ceramide. The latter is hydrolyzed by acid ceramidase to sphingosine and fatty acid, completing a cycle in which sphingosine can re-enter the biosynthetic machinery via the salvage pathway. This perpetual synthesis–remodeling–degradation loop enables cells to adapt their GSL landscape to developmental, physiological, and pathological demands.

### Regulation of GSLs

Leveraging single cell lipidomics and transcriptomics, a recent study has unveiled a decisive link between GSL composition and the very fate and identity of cells. In human dermal fibroblasts, distinct lipid compositional states, or “lipotypes”, were found to correlate with transcriptional subpopulations [33]. Strikingly, manipulating sphingolipid composition was sufficient to reprogram fibroblasts toward distinct phenotypic states, underscoring a causal role for GSL metabolism in cell fate transitions. In fact, a self-sustaining regulatory loop was identified, wherein globo-series GSL Gb4 positively modulates FGF2 signaling, while ganglio-series GSL GM1 inhibits it. In turn, FGF2 signaling reinforces globo-series biosynthesis, establishing a metabolic-transcriptional feedback circuit that maintains fibroblast heterogeneity. These findings highlight how GSL metabolism is tightly intertwined with signaling networks, offering a new lens through which to understand cellular plasticity, tissue organization, and possibly even therapeutic reprogramming strategies.

Epigenetic mechanisms such as histone acetylation also have been shown to orchestrate global changes in GSL profiles during neural development [34–36]. At the post-translational level, the enzymatic activity of glycosyltransferases is fine-tuned via modifications such as phosphorylation; for instance, phosphorylation inhibits sialyltransferase ST3GAL4 while activating B4GALNT1, thereby shifting the balance between gangliosides like GM1, GM2, and GD1a [37].

In addition to enzymatic regulation, GSL biosynthesis is influenced by the formation of multi-enzyme complexes in the Golgi, where sequential glycosylation steps are performed in a highly coordinated manner. The availability of nucleotide sugar donors and their transport across Golgi membranes also represents a rate-limiting checkpoint in this biosynthetic pathway [38, 39].

Intriguingly, GSLs are not static entities: their plasma membrane composition can change rapidly in response to local cellular cues, as stated earlier. Plasma membrane-associated glycosyltransferases such as sialyltransferases can locally remodel the GSL landscape, enabling rapid adaptation to environmental or developmental demands [32]. For example, the conversion of GM1 to GD1a at the cell surface is critical for axonal growth and neural repair. Resolving the source of nucleotide-sugars for glycosyltransferases to function at the cell surface remains an important matter. Furthermore, lysosomal fusion with the plasma membrane may transiently expose hydrolases to the extracellular interface. How these enzymes retain activity in neutral pH environments has only recently started to be revealed [40].

Together, these layers of regulation highlight how GSL composition, biosynthesis, and remodeling are tightly integrated with cellular state, signaling, and membrane dynamics, allowing GSLs to serve as both functional effectors and responsive regulators in diverse biological processes.

While these findings highlight the regulatory role of GSLs in physiological contexts, their dysregulation is equally evident in pathological settings. Distinct GSLs expression patterns have been observed across cancer types. For example, O-acetyl GD2 is overexpressed in neuroblastoma [41], GD1a in prostate cancer [42], and GD3 in breast cancer [43], underscoring their potential as both biomarkers and therapeutic targets.

### GSLs in physiology

The physiological significance of GSLs has been extensively characterized over the past decades, as described in Refs [3, 46]. and exemplified in gene knockout studies by a range of distinct phenotypic outcomes (Fig. 1b). Strikingly, inhibition of individual biosynthetic branches in mice often produces mild or no overt phenotypes, likely due to compensatory activity from parallel pathways. Indeed, combined GSL synthase gene knockout models generally show more severe phenotypes. For example, single knockouts of GM3 synthase, GD3 synthase, or GM2/GD2 synthase yields viable mice with near-normal lifespans and only subtle defects, such as insulin sensitivity, mild dysmyelination, or some axonal degeneration [47–49]. In case of double knockouts—removing both a- and b-series gangliosides—causes more pronounced consequences such as severe neurodegeneration, lethal audiogenic seizures, and early death, underscoring their complementary roles in CNS function [48, 50]. Disruption of the GlcCer synthase gene (UGCG), which catalyzes the first step in most GSL pathways, is even more severe, causing embryonic lethality or, in brain-specific knockouts, fatal neurodevelopmental defects within weeks [9, 51, 52].

Beyond the CNS, GSL depletion in proximal tubule epithelial cells (PTECs) alters protein reabsorption and xenobiotic uptake [53]. Loss of Gb3 reduces albumin and protein reabsorption but can protect against nephrotoxicity in acute kidney injury by limiting myoglobin and aminoglycoside uptake [53]. Gb3-deficient mice also show impaired immune responses during vaccination including reduced germinal center formation, reduced plasma and T follicular helper (TFH) cell differentiation, and defective antibody affinity maturation [54]. GSLs are also critical for intestinal epithelial integrity and nutrient absorption [55]. In inducible Ugcg^flox/Cre-villin mice, acute enterocyte-specific deletion caused rapid epithelial differentiation defects, impaired nutrient uptake, and death from malnutrition [56]. Similar essential roles are seen in skin barrier maintenance (keratinocyte-specific UGCG deletion) [57]. Loss of the B3GNT5 gene, abolishing lacto series gangliosides, causes variable postnatal outcomes ranging from normal early life to growth retardation, premature death, splenomegaly, lymphadenopathy, fur loss, obesity, reproductive defects, and impaired B-cell function with reduced numbers, absent germinal centers, and diminished proliferative capacity [58].

### GSLs in pathology

GSLs have physiological roles in vital organs such as kidney and intestine. What then about their pathological functions?

#### Cellular entry of pathogens and pathogenic factors

Some bacterial toxins (e.g., Shiga and cholera toxins) and animal viruses (e.g., polyomaviruses such as simian virus 40) exploit GSLs as cell surface receptors to gain access to host cells, and trigger cytotoxicity or infection [59, 60]. See below for a more detailed discussion on pathogenic GSL-binding proteins and their link to infectious disease.

#### Lysosomal storage disorders

Most lysosomal storage disorders are caused by defects in enzymes that degrade GSLs, which leads to GSL accumulation in lysosomes [39]. For example, Fabry disease is caused by mutations in the GLA gene that lead to deficiency in α-galactosidase A enzyme activity. As a result, Gb3 accumulates in lysosomes, which affects kidneys, heart, and blood vessels [61]. Gaucher’s disease is caused by deficiency in glucocerebrosidase, which results in accumulation of glucocerebroside in lysosomes, causing organ damage [62].

#### Neurodegeneration

GSLs are also linked to neurodegenerative diseases. For instance, dysregulation of GSL levels, e.g., glucosylceramide (GlcCer) or gangliosides, disrupts cellular processes, including lysosomal function, protein aggregation, and intercellular signaling, ultimately leading to neuronal damage and degeneration [63, 64]. Mutations in GBA1 that result in reduced glucocerebrosidase activity have been identified as the most common genetic risk factors for Parkinson’s disease. The resulting lipid accumulation perturbs autophagy-lysosome and mitochondrial pathways, promoting α-synuclein aggregation. Gangliosides also modulate amyloid-β (Aβ1–42) assembly [65]. GM1–Aβ(1–42) complexes can act as seeds for Aβ aggregation, although context-dependent neuroprotective effects of gangliosides in presence of the raft-enabling lipids cholesterol and sphingomyelin are also reported, thereby highlighting the importance of membrane composition.

#### Congenital disorders of glycosylation

Defects in GSL biosynthesis cause rare genetic diseases [66]. For example, GM3 synthase deficiency (*ST3GAL5*) leads to infantile-onset epileptic encephalopathy with sensory defects and changes in pigmentation [66, 67]. GM2/GD2 synthase deficiency (*B4GALNT1*) underlies spastic paraplegia type 26 with complex neurological features [66, 68]. Human genetics and functional studies confirm loss of complex gangliosides as causal in these neurodevelopmental syndromes.

#### Cancer

GSLs are dysregulated in multiple cancers, including skin, breast, colon, ovarian, lung, melanoma, and prostate [69]. GSLs modulate critical signaling pathways that control tumor growth, survival, and metastasis [69–73]. For example, in colon and breast cancers, Gb4 interacts with EGFR to activate ERK, promoting tumor proliferation [74]. GM2 mediates tumor cell migration through integrin-dependent activation of FAK and ERK, and its overexpression or exogenous addition enhances cell motility [75]. GM3 inhibits EGFR autophosphorylation through interactions between its sialic acid residue and lysine K642 [76], and induction of GM3 synthesis by valproic acid is sufficient to suppress cancer cell proliferation [77]. GD3 regulates Fas-mediated apoptosis in glioma by forming a signaling complex with PDGF-α and Yes kinase; disruption of this complex induces apoptosis and inhibits metastasis [78, 79]. Similarly, elimination of Gb3 in colon cancer cells by Gb3-synthase knockdown reduces cell migration, highlighting its role in invasiveness [80]. Moreover, GM2, GD3, and Gb3 are associated with increased tumor angiogenesis [72, 73]. Overall, GSLs contribute to cancer progression through multiple mechanisms, including modulation of receptor signaling, promotion of tumor growth, migration, and angiogenesis, which makes them key regulators in cancer biology and potential therapeutic targets.

#### Immune related pathology

In a recent study, age-associated upregulation of GD3 on senescent cells was shown to impair natural killer (NK) cell-mediated cytotoxicity by inhibiting degranulation, thereby enabling senescent cells to evade immune surveillance. Notably, therapeutic targeting of GD3-positive senescent cells in mice attenuates age- and disease-associated pathological changes, including fibrosis in the lungs and liver, as well as aberrant bone remodelling [81]. Moreover, high levels of GSLs in tumors also negatively affect dendritic cell differentiation and function, impairing their ability to present antigens and activate T cells [72, 82]. In a recent phase I clinical trial, the combination of eliglustat, a GSL synthesis inhibitor, and anti-PD-1 antibodies demonstrated improved efficacy in patients with advanced cancers [83]. This provides evidence that GSLs and checkpoint molecules could function together.

Research from the past decades has thereby uncovered a clear function of GSLs in various physiological processes and disease situations. The molecular mechanisms by which this occurs remain in many cases still unknown. While their structural diversity and metabolic regulation set the stage, it is in endocytosis that the functional impact of GSLs becomes most apparent where they orchestrate membrane trafficking processes that shape cellular communication and drive disease progression.

## Endocytosis as a functional platform: Clathrin-dependent and independent processes

The plasma membrane acts not only as a barrier but also as a responsive and adaptable interface. Through the process of endocytosis, it allows for the internalization of extracellular cargoes and orchestrates a wide array of essential cellular functions that range from nutrient uptake and growth factor signaling to the dynamic maintenance of specialized areas of the plasma membrane [84, 85].

The plasma membrane also acts as a barrier against extracellular insults. Nevertheless, many pathogens and pathogenic agents, including viruses, bacteria, and protein toxins, have evolved sophisticated mechanisms to subvert this barrier, exploiting host membrane components and endocytic processes to gain access to the intracellular environment. These sophisticated entry strategies highlight the plasma membrane’s dual role as both gatekeeper and gateway.

**Fig. 2:**
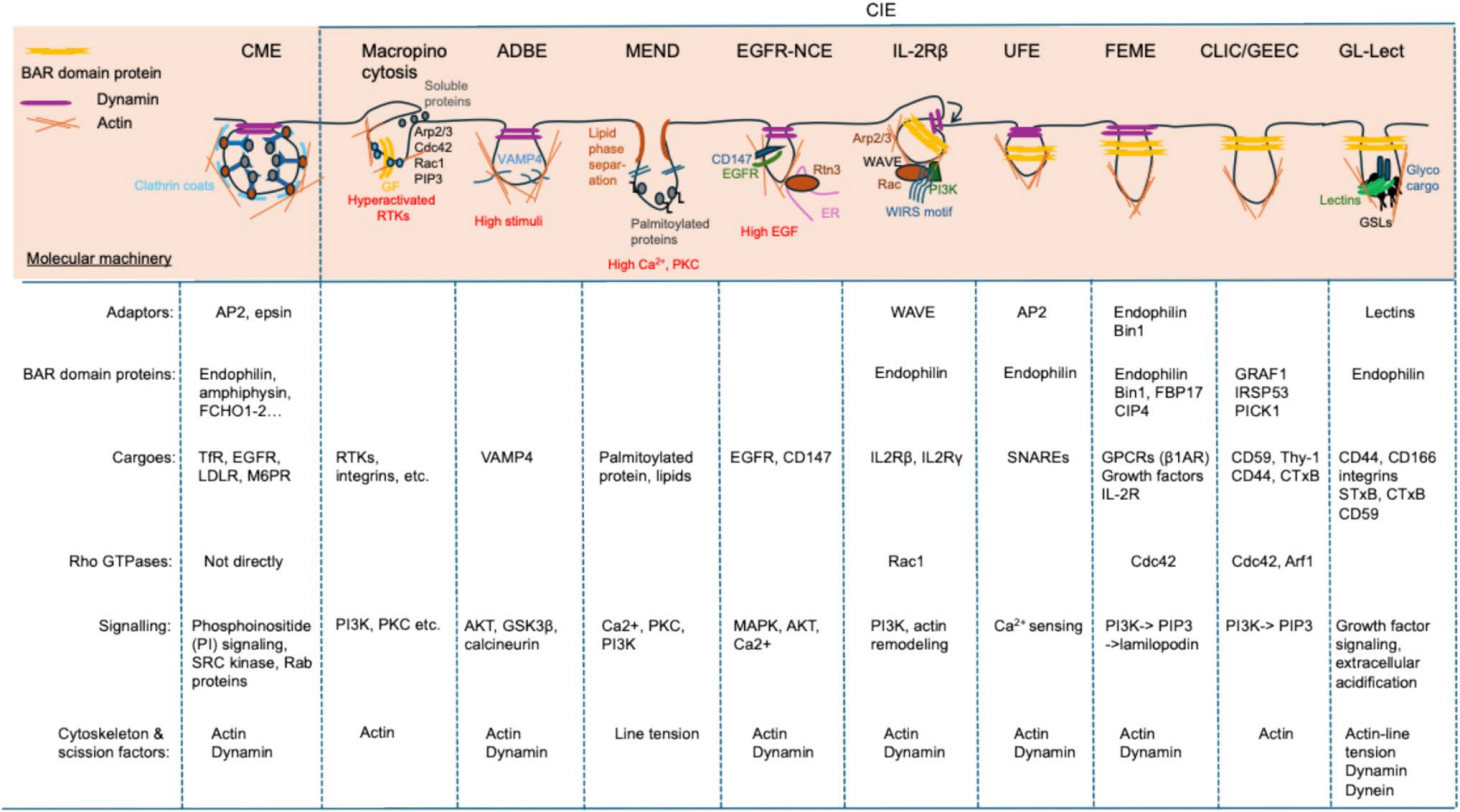
Comparison of clathrin-mediated endocytosis and various clathrin-independent endocytic (CIE) processes (adapted from Refs [88, 97].). Each endocytic process is illustrated with its characteristic endocytic pits and associated molecular machinery, including adaptors, BAR domain proteins, cargoes, Rho GTPases, signaling components, and cytoskeletal/scission factors. See text for details. The figure was prepared using Powerpoint

Clathrin-mediated endocytosis is by far the best characterized endocytic uptake mechanism [84–86]. Since its discovery in 1975, over 50 proteins have been identified to be part of the molecular machinery that generates the clathrin-coated endocytic vesicles [86]. The clathrin triskelion is recruited to the plasma membrane via cytosolic adaptor proteins (e.g., the tetrameric adaptor AP2), which bind to consensus signals (e.g., NPxY and di-leucine) located in the cytosolic tails of cargo receptor proteins (e.g., the transferrin receptor). Through its self-assembly properties and the recruitment of curvature-inducing factors such as epsins and BAR-domain proteins, clathrin shapes the formation of cargo loaded clathrin-coated pits. Their scission from the plasma membrane is executed by the GTPase dynamin, releasing clathrin-coated vesicles into the cytoplasm. The clathrin coat is then removed, and uncoated vesicles fuse into early endosomes (Fig. 2).

For decades, clathrin was regarded as the universal engine of endocytic pit formation. This view has since evolved, as it is now well established that blocking the clathrin pathway does not halt all endocytic uptake processes. Instead, several alternative clathrin-independent endocytosis (CIE) mechanisms remain active and continue to mediate efficient endocytic uptake [6, 87–89]. These CIE mechanisms appear to be highly context-specific and influenced by multiple factors including cargo sorting signals, plasma membrane lipid composition, actin cytoskeleton dynamics, glycosylation status of cargoes and GSLs, and extracellular clustering cues. No single molecular actor has yet been identified that would be shared by all CIE processes and that would not also be involved in the clathrin pathway. In this review, we therefore refrain from using the term “pathway” for the CIE processes. We favor a view according to which each individual CIE process needs to be characterized as to the elements of molecular machinery that are involved in cargo recruitment (e.g., glycans, lectins, actin, adaptors, flotillins), membrane bending (e.g., GSLs, actin, BAR domain proteins), and scission (e.g., dynamin, dynein, scaffolding proteins, actin) [90]. In many cases of CIE, the resulting uptake intermediates have short tubular morphologies [91–94]. This most likely reflects the absence of a spherical coat such that tubular endocytic pits are formed whose scission generates tubular endocytic carriers of somewhat variable length. Below we provide a brief panoramic overview of the possibly best characterized CIE processes (Fig. 2). Phagocytosis is not considered here, and the reader is referred to excellent reviews on this theme [95, 96].

### Macropinocytosis

Often activated by growth factors (e.g., EGF, PDGF, M-CSF, HGF, IGF), oncogenic signals (e.g., mutated Ras), or pathogen associated stimuli (e.g., bacterial effectors or viral entry), macropinocytosis relies on small GTPases (e.g., Rac1, Cdc42, Arf6, Rab5) and lipid signaling to drive actin-dependent membrane cup formation by plasma membrane enriched in PIP3 [89, 98]. Unlike phagocytosis, macropinocytosis preferentially engulfs fluid-phase cargo and is particularly prominent in immune cells and cancers, where it supports antigen surveillance and metabolic adaptation [89, 98–100].

### Massive Endocytosis (MEND)

This endocytic process is typically triggered by transient stress signals such as Ca^2+^ influx, PKC activation, or PI3K signaling, and preferentially occurs in actin-free regions of the membrane [88, 101, 102]. Mechanistically, the process is believed to rely on the coalescence of lipid-ordered nanodomains at the outer leaflet, which promotes negative curvature and inward buckling of the membrane, leading to the formation of endocytic pits. The driving force for membrane scission may be provided by line tension at domain boundaries, leading to vesicle release independently of dynamin [103].

### EGFR Non-Clathrin Endocytosis (EGFR-NCE)

EGFR uptake switches from clathrin-dependent endocytosis to CIE at high EGF concentrations, leading to lysosomal degradation and signal desensitization [104–106]. This process specifically requires mono-ubiquitination of EGFR and reticulon-3 mediated ER–plasma membrane contacts, which trigger localized Ca^2+^ signaling and mitochondrial metabolism to form tubular endocytic carriers [94, 107]. Other receptors like CD147/basigin are also internalized via this process [94].

### Activity-Dependent Bulk Endocytosis (ADBE) [108]

At neuronal synapses, activity-dependent bulk endocytosis is triggered under conditions of intense neuronal stimulation, when calcium influx in synaptic terminals and massive vesicle exocytosis overwhelm clathrin-mediated and ultrafast endocytosis (see below). This process is regulated by calcium signaling through calcineurin and kinases like Cdk5, GSK3β, and AKT, which control key proteins like dynamin-1 and syndapin-I. It is also dependent on the acidification of endosomes by the V-type H + -ATPase [109]. Activity-dependent bulk endocytosis provides a rapid way of retrieving synaptic vesicle membranes after neurotransmitter release.

### Fast Endophilin-Mediated Endocytosis (FEME)

FEME is not constitutively active but is triggered by the activation of specific cell surface receptors such as GPCRs, receptor tyrosine kinases, cytokine receptors, and axon guidance receptors (PlexinA1 and ROBO1) [93, 110]. In resting cells, endophilin clusters transiently on the plasma membrane, involving signaling molecules like Cdc42, PI(3,4)P₂, and lamellipodin [111]. Upon receptor activation, cargoes are sorted into these clusters, leading to the rapid formation of tubular FEME carriers with the help of Bin1, actin polymerization, dynamin, and friction-driven scission [93, 112]. FEME is regulated by phosphoinositides and kinases like Src, LRRK2, Cdk5, and GSK3β. In fact, FEME is negatively regulated by Cdk5 and GSK3β, which phosphorylate key residues on cargo adaptors, dynamin, and dynein, thereby preventing endophilin and Bin1 binding [110]. FEME is prominent at the leading edge of migrating cells and plays roles in cellular processes requiring fast internalization, such as stress responses and receptor hyper-stimulation. FEME is also hijacked by pathogens like cholera and Shiga toxins. Importantly, many of these cargoes can enter cells through other endocytic processes (see below). In contrast, β1-adrenergic receptor relies entirely on FEME [113].

### Interleukin-2 Receptor (IL-2R) Uptake

In T cells, IL-2R is mainly internalized via FEME [93]. However, isolated IL-2Rβ or γ chains in non-immune cells use a distinct process involving WAVE1 complex, a regulator of actin nucleation—through WIRS motifs (WAVE Regulatory Complex Interacting Receptor Sequences) in the cytoplasmic tail of IL-2Rβ [114, 115]. Clustering of IL-2Rβ chains triggers local actin protrusion via Arp2/3, forming small spherical endocytic pits [114, 116, 117]. The uptake process is further facilitates by PI3K signaling, local production of PI(3,4,5)P3, and Rac1-mediated activation of WAVE1 and PAK ((p21-activated kinases) proteins [116, 117]. Scission of these carriers depends on endophilin A2, cortactin, and dynamin-2.

### Ultrafast Endocytosis (UFE)

Ultrafast endocytosis is a rapid synaptic vesicle recycling process that retrieves membrane within ~ 50–100 ms immediately after exocytosis. This process is prominently observed in motor neurons and hippocampal neurons. It is mechanically triggered: Fusion of synaptic vesicles compresses the plasma membrane, locally releasing tension leading to the buckling of the membrane and the nucleation of endocytic pits at the edge of active zones [118–120]. These pits rapidly pinch off with the help of actin and dynamin [121]; clathrin then comes later on endosomes, not at the cell surface. These features explain how nerve terminals maintain membrane balance and sustain transmission during bursts of activity.

### Caveolar endocytosis

Caveolae are flask-shaped plasma membrane invaginations that are formed by membrane-associated caveolins (CAV1–3) and soluble cavins [87, 122–124]. While caveolae were initially portrayed as endocytic carriers [125–128], later work has called this notion into question [129]. It has indeed been shown that presumed cargoes of caveolar endocytosis are taken up equally well or even better when caveolae are removed from cells [130]. The consensus is now that caveolae are predominantly mechanoprotection, mechanotransduction, and mechanosignaling devices [129, 131]. The endocytosis of caveolar components occurs, and is mediated by dynamin, and negatively regulated by the ATPase EHD2 [132–134]. This likely serves to reassemble caveolae after their flattening and to set their levels at the plasma membrane.

### Flotillin-assisted endocytosis

Flotillin-1 and −2 are membrane nanodomain-associated proteins that have been implicated in CIE, particularly in the uptake of GPI-anchored proteins and certain signaling receptors [135–137]. Whether flotillins primarily contribute to pre-endocytic cargo clustering or membrane organization, or act directly as core drivers of membrane budding remains an active field of investigation. Recent findings underscore flotilin-assisted endocytosis as a safeguard mechanism, preventing premature plasma membrane rupture and necroptotic cell death [138], and favoring drug uptake by cancer cells [139] and cholesterol absorption [140].

### CLIC/GEEC

This endocytic process involves the formation of short, sometimes crescent-shaped uncoated tubulovesicular structures that have been termed clathrin-independent carriers (CLICs) [141]. CLICs subsequently mature into glycosylphosphatidylinositol (GPI)-anchored protein-enriched early endocytic compartments (GEECs) [87]. GEECs then fuse with sorting endosomes in a process that is dependent on the activity of Rab5 and PI3K [142]. The CLIC/GEEC process is responsible for the uptake of the ganglioside-binding B-subunit of cholera toxin (CTxB) [141], glycosylphosphatidylinositol-anchored proteins (such as CD59, folate receptor, or CD90/Thy-1) [91, 143], transmembrane proteins (e.g., CD44, CD98) [91], and for a significant portion of fluid-phase endocytosis [91]. CLIC/GEEC endocytosis is a major driver of rapid membrane recycling; this dynamic turnover supports homeostasis and contributes to plasma membrane repair in contexts such as toxin-induced lesions. CLIC/GEEC endocytosis is regulated by the small GTPases Arf1 and CDC42 [143, 144], the GTPase activating factor and BAR domain protein GRAF1 [145], the actin nucleation factor ARP2/3 [146], and the BAR domain proteins IRSp53 and PICK1 [147]. CLIC/GEEC endocytosis is dynamin-independent [144], involved in processes like plasma membrane tension regulation [148], organ development [149], cancer progression [150], and is exploited by certain viruses for cellular entry [151, 152].

## Glycolipid-lectin (GL-Lect) driven endocytosis

GSLs play a central role in an endocytic mechanism that has been termed GL-Lect driven endocytosis [6]. Considering the GSL focus of the current review, GL-Lect driven endocytosis will be introduced here in further detail.

The concept of GL-Lect driven endocytosis goes back to GSL-binding bacterial toxins and viruses, notably cholera and Shiga toxins and polyomaviruses such as simian virus 40 [153]. These were described as having membrane active properties, i.e., the capacity to change membrane order through oligovalent GSL binding and clustering [154–158]. The patch of membrane that these toxins and viruses are associated with thereby gains characteristics of raft-type nanodomains [159–162], i.e., enrichment in raft fabric (here: GSLs [163, 164]), high membrane order [154–158], and functional roles in endocytic trafficking [155, 165].

**Fig. 3:**
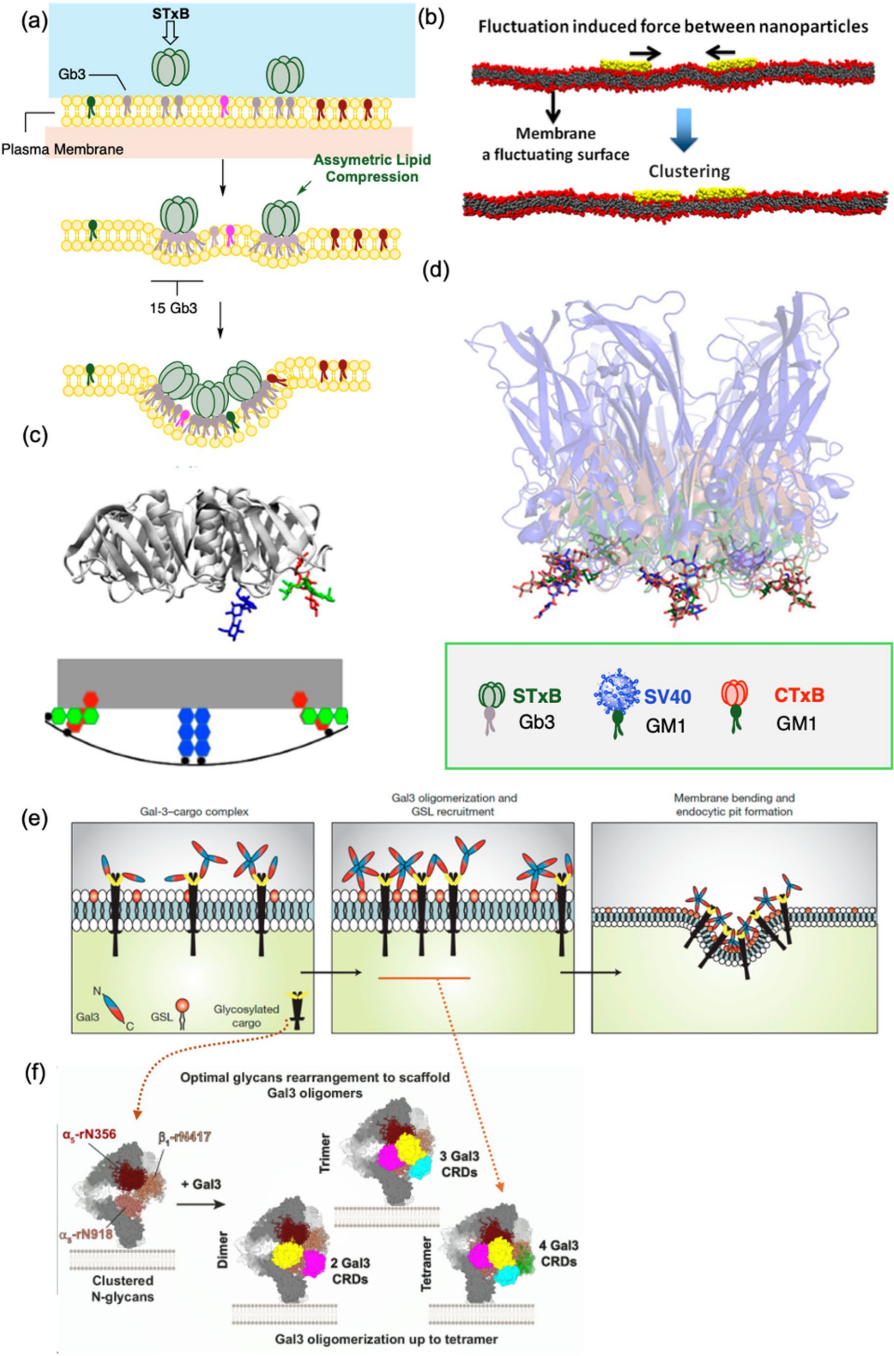
Building endocytic pits via glycolipid–lectin interactions and membrane remodeling. **a** Mechanism of GSL Gb3 reorganization and asymmetric compressive stress induced by STxB, leading to negative membrane curvature and endocytic pit formation (inspired from Ref [175].). The figure was prepared using Powerpoint. **b** Nanometric membrane inclusions such as STxB suppress thermally excited membrane fluctuations at their binding sites and in adjacent patches when separated by distances comparable to their size. This suppression creates an attractive force between inclusions (reproduced from Ref. [170].). **c** Structural view of STxB (side view) with three Gb3 glycans (blue, green and red) in corresponding binding sites. For simplicity, binding sites are shown only for one of the monomers. Peripheral sites 1 and 2 (red, green) require upward membrane bending to engage, while site 3 (blue) faces down under the protein, promoting negative curvature (reproduced from Ref [157].). **d** Overlay of STxB–Gb3, cholera toxin B-subunit (CTxB)–GM1, and SV40 VP1–GM1 complexes reveals a conserved spatial arrangement of GSL receptor-binding sites despite lack of sequence similarity. These proteins fold such that the conserved GSL binding site 2 is presented with the same geometry in space (the glycans of the three structures overlay), suggesting convergent evolution for the same function: to generate negative membrane curvature for building endocytic pits for clathrin-independent endocytosis (reproduced from Ref [175].). **e** Gal3-driven endocytosis: sequential steps from glycosylated cargo recognition, Gal3 oligomerization, and glycosphingolipid recruitment to drive membrane bending and tubular endocytic pit formation (reproduced from Ref [92].). **f** Optimal glycan rearrangement scaffolding Gal3 oligomers up to tetramers (reproduced from Ref [181].). Together, these panels depict how oligomeric lectin–glycan interactions orchestrate nanoscale clustering, membrane deformation, and cargo internalization without the clathrin coat

The mechanisms by which GSL reorganization into raft nanodomains is translated into endocytic site construction have been particularly well studied for Shiga toxin (STx). STx is secreted by pathogenic strains of *E. coli*, and its presence has been linked to pathological manifestations leading to life threatening disease, notably hemolytic uremic syndrome (HUS) [59, 166]. STx is a prototypical AB5 toxin, composed of a catalytically active A-subunit and a homopentameric B-subunit (STxB). STxB mediates binding to up to 15 globotriaosylceramide (Gb3) molecules on endothelial and other cells [167] and directs the intracellular trafficking of the A-subunit from the cell surface via the Golgi apparatus to the endoplasmic reticulum and the cytosol [155, 156, 168]. Remarkably, interaction with Gb3 not only allows for STx to bind to cells. STxB-driven reorganization of Gb3 initiates the formation of narrow tubular endocytic pits that are emblematic of its clathrin-independent uptake into cells (Fig. 3a) [155].

This finding raises a central question: how is membrane curvature generated in the absence of the conventional clathrin coat? This question has been addressed through a combination of molecular dynamics simulation and chemical cell biology exploration. Remarkably, STxB homopentamers remain monodisperse in solution but undergo efficient clustering upon binding to the plasma membrane [169]. Based on dissipative particle dynamics simulations*,* it was suggested that this clustering emerges from the suppression of thermally induced lipid protrusion fluctuations between adjacent tightly membrane-bound STxB molecules [170]. This event generates an attractive force in the range of several nm, effectively pulling neighboring Gb3 receptors into nanoscopic clusters (Fig. 3b). This theoretical prediction has been experimentally tested using synthetic Gb3 analogues with flexible polyethylene glycol (PEG) linkers between globotriose glycan and ceramide, which revealed reduced STxB clustering and inhibition of endocytosis, likely because the suppression of lipid protrusion fluctuations diminished with increasing flexible PEG linker length [170, 171]. Furthermore, grazing incidence X-ray diffraction studies using the PEGylated Gb3 species led to the observation that STxB induces lipid compression specifically within the exoplasmic (outer) leaflet of the bilayer to which it is bound [158]. This creates an asymmetric compressive stress and promotes inward (negative) membrane curvature. The unique geometry of STxB’s Gb3-binding sites 1 and 2 further amplifies this effect: to fit the carbohydrate headgroups into these binding pockets that sit at the periphery of the ring-shaped pentamer above the normal plane of the membrane, the membrane must bend up toward the protein, adding a geometric constraint that favors negative membrane curvature (Fig. 3c) [157].

Together, asymmetric compressive stress and receptor geometry orchestrate the formation of narrow, tubular endocytic pits without the need for traditional coat proteins. Intriguingly, this mechanism is not unique to STxB. The B-subunit of cholera toxin (CTxB), which is structurally similar to STxB despite the lack of sequence similarity [172], interacts with up to five GM1 molecules as the cellular receptor and induces tubular membrane invaginations [163, 165, 173, 174], similar to STxB. Furthermore, similar curvature generation capacity has been demonstrated for the GM1-binding VP1 capsid protein from simian virus 40, pointing to a convergent evolutionary solution for remodeling membranes via lipid–protein cooperativity (Fig. 3d) [6, 153, 165, 169, 175, 176].

The structural characteristics of the acyl chains on Gb3 also play a pivotal role in the induction of membrane curvature [155]. Specifically, unsaturated acyl chains in Gb3 are essential for the formation of tubular membrane invaginations. In contrast, fully saturated acyl chains render the nanodomains assembled by STxB too rigid to be bent. The acyl chain flexibility endowed by unsaturation is critical for the biological activity of STxB, facilitating both efficient endocytic uptake and intracellular trafficking. A similar requirement for membrane rigidity has been observed for CTxB, where trafficking efficacy is significantly higher with monounsaturated GM1 species compared to their saturated counterparts [177]. Interestingly, CTxB also induces GM1 co-clustering with the GPI-anchored protein CD59 in an acyl chain saturation sensitive manner [163]. Together, these findings underscore the role of acyl chain diversity in trafficking processes driven by oligomeric GSL-binding lectins.

Tubular endocytic pits are recognized via the N-BAR domains of cytosolic endophilin-A2 [178, 179]. This interaction introduces friction that slows lipid diffusion, thereby preparing the tubule for scission under pulling forces generated by the molecular motor dynein [112, 178, 180]. In the case of Shiga toxin, this friction-driven scission [112] acts in concert with actin remodeling and dynamin activity to promote the release of tubular endocytic carriers from the plasma membrane [103, 155, 178]. Friction-driven scission likely also operates in GL-Lect driven endocytosis of cellular proteins such as integrins [40, 181] and the amino acid transporter CD98 and CD147/basigin [182].

Indeed, endogenous cellular lectins from the galectin family have also been shown to drive GSL-dependent membrane bending for CIE [92]. Galectin-3 (Gal3), for instance, binds as a monomer to glycosylated cargo proteins such as CD44 and α5β1 integrin. Gal3 then oligomerizes, forming multivalent interactions with both the cargo and GSLs [181]. This cooperative assembly induces localized curvature, driving the formation of tubular endocytic pits that undergo friction-driven scission to generate CLICs [40, 92, 181]. Gal3 thereby links cargo recognition via N-glycans to curvature generation via GSLs (Fig. 3e and f).

A similar mechanism of cargo clustering and membrane remodeling has been observed with galectin-8 for the GSL and endophilin-A3 dependent uptake of CD166/ALCAM, a tumor-associated adhesion molecule [183], and with Gal3 for the uptake of CD98 and CD147/basigin [182]. Collectively, these studies define the GL-Lect model of endocytosis [6, 153, 169], a finely tuned mechanism whereby oligomeric lectins translate membrane composition and cargo glycosylation into the construction of endocytic pits.

## Endocytic trafficking and polarized secretion

What, then, is the intracellular fate of glycoproteins that are internalized by GL-Lect driven endocytosis? Strikingly, several well-characterized cargo proteins entering via this process exhibit highly polarized localization patterns, which are integral to their specific cellular functions. Emerging evidence indeed places GL-Lect driven endocytosis at the heart of spatially regulated intracellular trafficking, linking it directly to fundamental polarity-dependent processes. These include: (i) directional β1 integrin localization to the leading edge of migrating cells to sustain persistent motility, (ii) lactoferrin transcytosis across intestinal enterocytes, (iii) sealing zone remodeling in osteoclasts for bone resorption, (iv) protein reabsorption in proximal tubule epithelial cells of the kidney, and (v) the localization of LAT to immunological synapses. Together, these roles highlight the potential of GL-Lect driven endocytosis in orchestrating membrane trafficking across diverse physiological landscapes—beyond mere uptake, this mechanism contributes to cell polarity, intercellular communication, and organ-specific functions.

### Leading edge polarity in migrating cells

Highly polarized migrating cells exhibit a distinct front–rear polarity that is essential for directional movement. A central component of this process is the dynamic regulation of integrins, which are heterodimeric α/β transmembrane receptors that mediate adhesion to the extracellular matrix and couple it to the actin cytoskeleton [184]. Among them, α5β1 integrin plays a pivotal role in orchestrating directional migration by maintaining its enrichment at the leading edge of cells [185]. Strikingly, it has been found that the conformational state of α5β1 integrin dictates its endocytic route: the ligand-bound active form is internalized predominantly via clathrin-dependent endocytosis in relation to focal adhesion kinase (FAK) activation [186], whereas the inactive, non-ligand-bound form is internalized predominantly by GL-Lect driven endocytosis [181]. It has indeed been shown that only the bent-closed non-ligand-bound form of α5β1 integrin nucleates Gal3 oligomers, and that only oligomeric Gal3 interacts with GSLs [181]. The molecular reasons for this surprising finding remain yet to be identified.

**Fig. 4:**
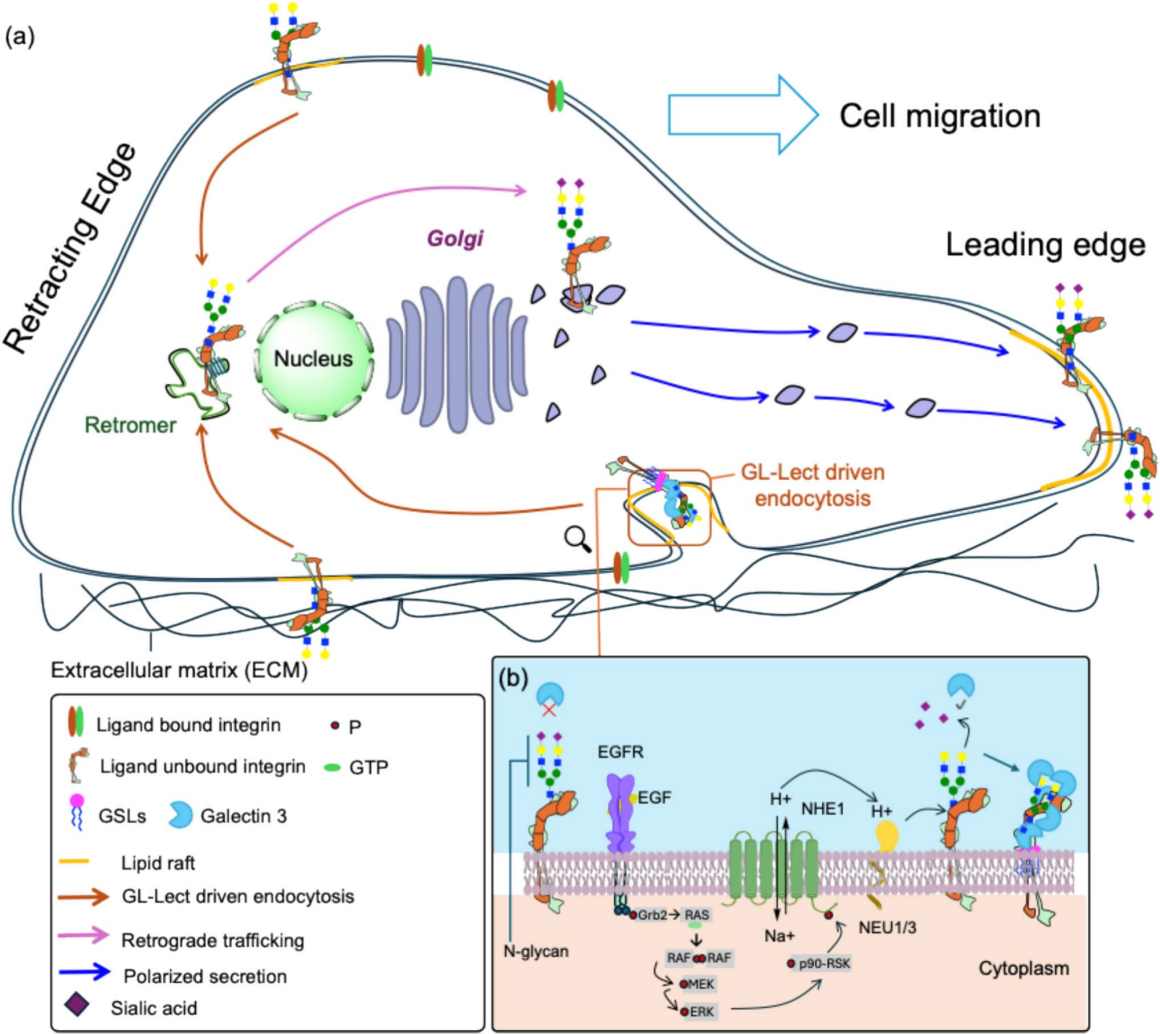
GlycoSwitch-mediated regulation of integrin trafficking and signaling during cell migration (adapted from Ref [97, 187].). **a** Schematic representation of retrograde trafficking in migratory cells. During directional migration (indicated by the thick arrow), the cell exhibits two distinct domains: the retracting rear and the leading edge. The Golgi apparatus is polarized toward the leading edge, supporting polarized secretion. Only non–ligand-bound bent-closed α5β1 integrin undergoes GL-Lect driven endocytosis and retrograde trafficking to the Golgi. Here, its glycan profile is reset before being re-secreted in a polarized manner to the leading edge. Topology note: The N-glycans on α5β1 integrin are extracellular/luminal. Figure elements are not drawn to scale. **b** Regulation of GL-Lect driven endocytosis. Upon triggering by EGFR, NHE1 promotes proton efflux which lowers extracellular pH, thereby activating NEU1/3-mediated removal of α2–6 linked sialic acids from N-glycans on α5β1 integrin. This enables Gal3 binding and oligomerization, promoting endocytic uptake and retrograde trafficking of the desialylated cargo. The activity of NHE1 is controlled through the MAPK pathway*.* The figure was prepared using Powerpoint

Following its GL-Lect driven uptake, bent-closed inactive α5β1 integrin is transported via the retrograde trafficking route to the Golgi apparatus, to then undergo polarized secretion to the leading edge (Fig. 4a) [181, 185]. Disruption of retrograde transport results in α5β1 integrin mislocalization and severely compromises persistent cell migration. In contrast, enhancement of front–rear polarity correlates with increased efficiency of retrograde trafficking, thereby reinforcing the spatial positioning of α5β1 integrin at the front of migrating cells [185].

Recent work has revealed an additional layer of complexity in α5β1 integrin regulation, showing that its internalization by GL-Lect driven endocytosis is dynamically modulated by extracellular cues such as EGF [40]. EGF triggers the activity of the sodium-proton antiporter NHE1, leading to extracellular acidification and activation of cell surface neuraminidases (Fig. 4b). Neuraminidase-catalyzed removal of α2–6 linked sialic acids from α5β1 integrin (and other cell surface glycoproteins) enables Gal3 binding and GL-Lect driven endocytosis. Internalized α5β1 integrin is then transported to the Golgi, where its glycan profile is reset to support polarized redistribution during cell migration, as described above. This regulatory cycle — termed the GlycoSwitch — highlights glycosylation as a reversible regulatory mechanism that fine-tunes integrin trafficking and sustains front–rear polarity [187, 188].

### Lactoferrin transcytosis in enterocytes

Enterocytes, the absorptive epithelial cells of the small intestine, exhibit a high degree of polarization, with distinct apical and basolateral membrane domains that support directional transport of nutrients and macromolecules [189–191]. A key physiological function of these cells is transcytosis, which is the endocytic uptake of cargo at the apical membrane, followed by intracellular trafficking and targeted release at the basolateral surface, ultimately delivering cargo to the bloodstream [192]. The neonatal Fc receptor remains one of the most well-characterized mediators of transcytosis [193]. Recent findings reveal that lactoferrin, a multifunctional breast milk–derived glycoprotein best known for its roles in iron transport, antimicrobial defense, and immune modulation, is a Gal3–dependent cargo that undergoes GL-Lect driven transcytosis in enterocytes [56]. Endocytosis of Gal3 and lactoferrin was indeed strongly impaired upon depletion of GSLs, in agreement with the GL-Lect model. Moreover, LTF trafficking was critically dependent on Gal3 expression, and ultrastructural studies revealed the localization of Gal3 within tubular endocytic CLIC structures. This case highlights how GL-Lect driven endocytosis operated in a tissue-specific context. Open questions remain about whether other intestinal galectins, such as the abundant galectin-4, can substitute for Gal3, which additional transcytotic cargoes might be regulated by the GL-Lect process, and whether transcytosis involves retrograde trafficking via the Golgi apparatus.

### Osteoclast sealing zone — A pH-regulated niche for GL-Lect driven endocytosis

In osteoclasts, bone resorption depends on acidification of the resorption lacuna (or sealing zone), a process driven by V-ATPase activity and Na⁺/H⁺ exchangers. Conversely, osteoblast differentiation and mineralization are highly pH-sensitive, with alkaline conditions favoring bone formation [194]. Recent evidence has revealed that Na⁺/H⁺ exchangers, neuraminidases, and Gal3 are all required to maintain the sealing zone, suggesting a key role for GL-Lect driven endocytic trafficking and the GlycoSwitch in this process [40], possibly by ensuring the continued relocalization of critical adhesion factors that is required in dynamically remodeling sealing zones. Of note, sialidosis patients with defective neuraminidase exhibit symptoms that are consistent with defects in bone remodelling [195], and the knockout of genes coding for galectin-1, Gal3 and galectin-8 lead to bone phenotypes [196–198].

### Protein reabsorption in proximal tubule epithelial cells

Proximal tubule epithelial cells (PTECs) of the kidney play a vital role in maintaining protein homeostasis by retrieving proteins from the urinary filtrate and returning them to the bloodstream [199]. This reabsorption process prevents the loss of essential plasma proteins such as albumin. Very recently it was shown that the depletion of the GSL Gb3 significantly affects reabsorption [53]. In the absence of Gb3, the uptake of proteins like myoglobin and of xenobiotics such as gentamicin—both toxic to proximal tubules—is also inhibited, thereby preserving renal structure and function in conditions like rhabdomyolysis and during aminoglycoside treatment [53]. At the molecular level, this reabsorption function is primarily mediated by the multiligand endocytic receptors megalin (LRP2) and cubilin, which are abundantly expressed on the apical surface of PTECs, facing the tubular lumen. LRP2/megalin is a glycoprotein whose sialylation is strongly increased in neuraminidase-1 knockout mice [200], suggesting that it could be regulated by the GlycoSwitch. In line with this, strong genome-wide association study (GWAS) signals for glomerular filtration trait (estimated rate) are found in NEU1 [201].

### Immunological and neuronal synapses

**Fig. 5:**
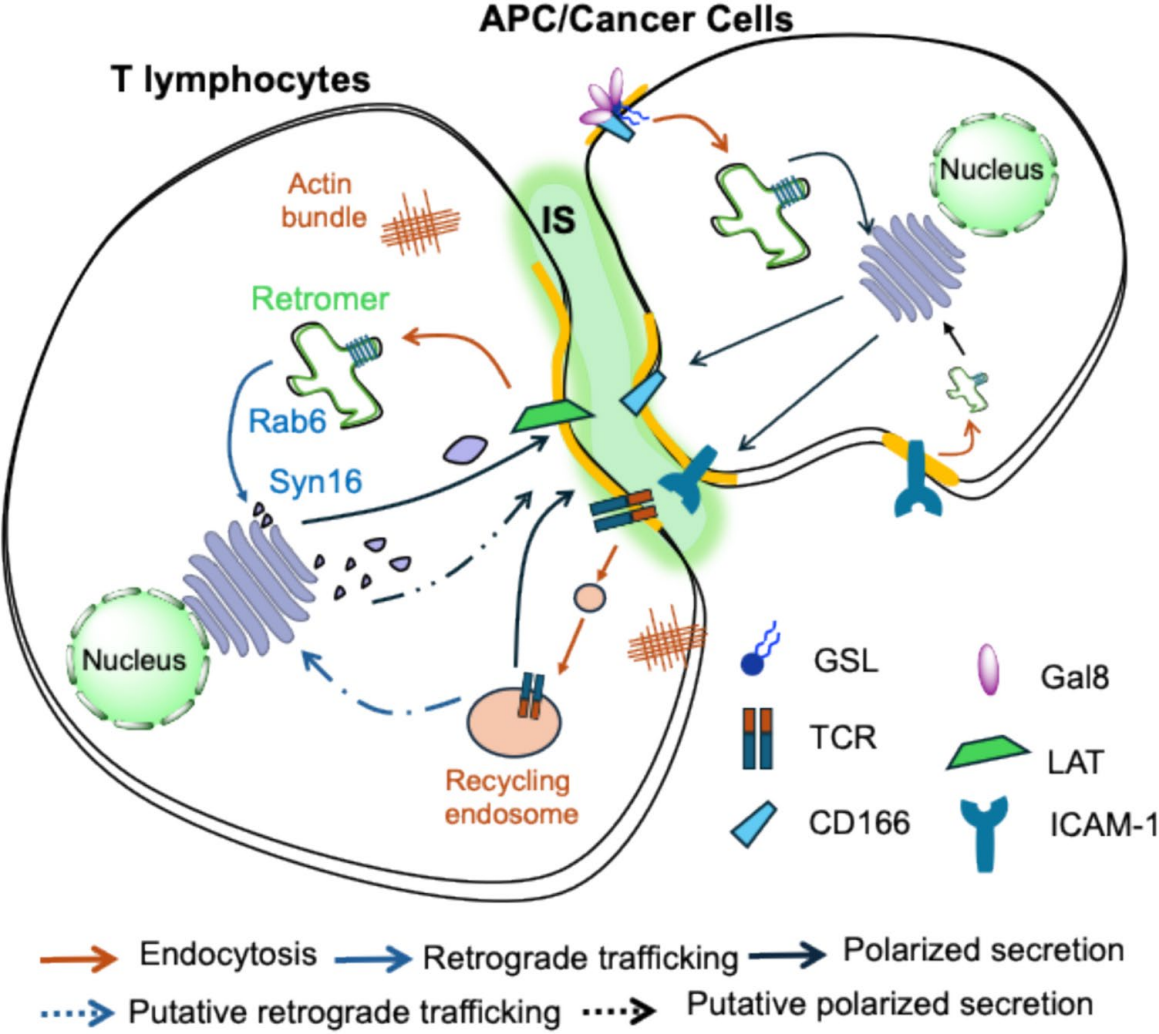
Retrograde trafficking and endocytic dynamics at the immunological synapse (adapted from Ref [97].). T-cell receptor (TCR) activation in the presence of antigen-presenting cells (APCs) induces formation of specialized membrane domains termed immunological synapses (IS), where the linker for activation of T cells (LAT) localizes in a polarized manner to organize the signaling complex. The Golgi apparatus is localized toward the IS to support polarized secretion. LAT undergoes Rab6, retromer, and syntaxin-16–dependent retrograde trafficking to the Golgi, enabling subsequent polarized delivery to immunological synapses—a loop essential for efficient T-cell activation [202, 207]. APC-derived cargoes such as CD166 [183] and ICAM-1 [208] have been reported to be internalized via CIE and to undergo retrograde transport to the Golgi for polarized redistribution at immune synapses [208]. The figure was prepared using Powerpoint

In T lymphocytes, activation of the T-cell receptor leads to the formation of the immunological synapse, a highly polarized membrane interface critical for antigen recognition and signaling. A central player in this process is LAT (Linker for Activation of T cells), a scaffolding adaptor that orchestrates signalosome assembly. Recent findings have shown that LAT undergoes Rab6, retromer, and syntaxin-16–dependent retrograde trafficking to the Golgi, followed by its targeted secretion to the immunological synapse (Fig. 5) [202]. LAT and other key signaling components, including the T-cell receptor, are known to associate with lipid raft nanodomains [203], and it was recently reported that the T-cell receptor is internalized via a CIE process [204]. Whether this involves galectins and GSLs as per the GL-Lect mechanism and whether CIE for all these proteins couples to retrograde trafficking and polarized secretion to the immunological synapse remains to be tested directly.

A parallel mechanism might exist at the neuronal synapse. In *C. elegans*, glutamate receptors are preferentially localized to dendrites, where their synaptic function relies on efficient retrograde trafficking [205]. Both clathrin-dependent and clathrin-independent endocytic processes contribute to their turnover [206]; however, the specific endocytic route that intersects with retrograde trafficking remains unresolved at this stage.

## Molecular unknowns and the need for advanced chemical tools in GSL research

Despite the critical roles of GSLs in numerous cellular pathways in both physiological and pathological contexts, they remain significantly underexplored compared to proteins and nucleic acids. Diseases such as lysosomal storage disorders or cancers, are associated with altered GSL expression. Notably, a prioritization of cancer-associated surface antigens identified four GSLs among the 75 top candidates, with GD2 ranking among the top 12 most clinically relevant targets in oncology [209]. Yet, the potential of GSL-targeted therapies remains largely untapped due to longstanding challenges in studying these structurally diverse and biosynthetically non-templated glycolipids.

Research on GSLs spans many disciplines from chemistry and biophysics to cell biology and immunology, to name a few. The small size of GSLs and their well-defined chemical architecture make them attractive substrates for synthetic derivatization, but their study has lagged due to the difficulty in obtaining dedicated tools for probing GSL–protein and GSL–GSL interactions. Unlike protein–protein or protein–nucleic acid interaction studies for which many highly specific, sensitive, and user-friendly techniques are available (e.g., proximity ligation or chromatin immunoprecipitation), the GSL research field lacks platforms that would be used in a similarly widespread manner.

It is therefore of note that recent advances in chemical biology have sparked a new wave of tool development for probing GSLs biology, particularly in the context of membrane organization and trafficking [210]. Fluorescently labeled GSL analogs such as BODIPY-labeled lactosylceramide (LacCer) and Gb3 have been synthesized to track their intracellular distribution in sphingolipid storage disorders and to monitor membrane organization [12, 211–214]. Additionally, photoaffinity-labelled probes derived from GM1, GM2, and GA2 have enabled the mapping of GSL–protein interaction networks [215–221]. A recent study introduced bifunctional, clickable photoaffinity gangliosides that are delivered directly to the plasma membranes of cultured human cells and then used to capture ganglioside–protein interactomes [221]. It has been demonstrated that interactome composition depends on cell type and on ganglioside structure and labeling site, and enriched interactions with transporters and adhesion proteins (e.g., integrins, cadherins, laminins) were uncovered. These tools incorporate photoreactive groups and clickable tags for enhanced versatility. A persistent challenge in this field remains in the need of maintaining the structural and functional integrity of GSLs. Many labeling strategies involve modifications to glycan headgroups or of ceramide acyl chains, and it cannot be excluded that membrane partitioning or protein interaction behaviors are affected [12]. In this context, the Gb3–STxB interaction offers a particularly instructive case. In phase-separated giant unilamellar vesicles (GUVs), fluorescent labeling of the fatty acid chain caused Gb3 to localize predominantly in the liquid-disordered phase, disrupting its native association with the liquid-ordered phase and abrogating effective STxB binding [12].

More refined approaches have therefore been developed. One strategy involves introducing a fluorophore via an oligoethylene glycol spacer at the 2’-OH position of the middle galactose in the Gb3 headgroup—identified through crystallography and trisaccharide binding studies as uninvolved in STxB interaction [213]. This design preserves binding while allowing control over the ceramide structure, enabling studies of how fatty acid (un)saturation influences Gb3 phase preference—e.g., shifting localization from liquid-ordered (C24:0) to liquid-disordered (C24:1).

While they are highly elegant, such headgroup modification strategies come with important caveats. First, published findings are derived from cell-free model systems, which may not fully recapitulate the complexity of cellular membranes. Translation to biological contexts requires caution, as the spatial organization, protein crowding, and lipid diversity in cells could significantly alter GSL behavior. Second, although the 2'-OH position is structurally tolerant in Gb3 for STxB binding, this approach may not be generalizable to other GSLs and other Gb3 interacting partners, where glycan architecture varies widely and often directly mediates protein recognition. Moreover, synthetic access to such precisely modified glycan structures remains technically demanding and is far from routine.

Taken together, these observations reinforce a key principle: preserving the native structure of both the glycan and ceramide domains is crucial for functional GSL probe design. Continued development of minimally perturbing, positionally selective labeling strategies will be essential for achieving the most biologically meaningful insights into GSL-mediated membrane dynamics and trafficking.

### Possible design principles for next-generation GSL tools

A central challenge in the development of next-generation GSL tools lies in introducing chemical modifications such as imaging or affinity tags without compromising the functions of these highly specialized membrane lipids. As discussed earlier, both the glycan headgroup and the ceramide tail are structurally and functionally sensitive, making them generally unsuitable targets for derivatization. However, two examples have opened up new design possibilities by identifying a chemically permissive region within the GSL molecule: the glycan–ceramide junction [165, 170]. In one study, Gb3 analogues were synthesized that incorporated PEG linkers at the junction between the globotriose headgroup and ceramide backbone [170]. Increasing PEG linker length disrupted Gb3's ability to suppress membrane fluctuations, an essential prerequisite for STxB clustering and subsequent endocytosis. However, short linkers allowed to retain both, high-affinity binding by STxB and the capacity of STxB to induce tubular membrane invaginations, suggesting that this junction is a chemically permissive site—provided modifications remain within a narrow range of lengths.

Similar insights arise from another study, here on GM1—the canonical receptor for cholera toxin. When the glycan moiety of GM1 was linked to a phospholipid via a phosphate-containing junction (rather than its native ceramide), this phospholipid-based GM1 variant was still efficiently recognized by the receptor-binding B-subunit of cholera toxin, which also induced tubular membrane invaginations for its own uptake into cells with this lipid as its cellular receptor [165]. Furthermore, phospholipid-based GM1 was also able to support viral entry, as demonstrated in the context of polyomavirus SV40 infection [165].

Taken together, these studies outline a core design principle: the glycan–ceramide junction as a tunable hotspot. With precise chemical engineering, particularly using short, flexible linkers or alternative lipid anchors, it may be possible to generate GSL probes that maintain essential biophysical and functional properties, while enabling enhanced synthetic accessibility and modularity. Such advances are expected to be instrumental for building the next generation of minimally perturbing tools to probe GSL-mediated membrane processes in living systems.

### Biotherapeutic applications of GSL-targeting strategies

Antibodies have revolutionized cancer therapy, particularly as antibody–drug conjugates for targeted tumor cell killing, and as blockers of inhibitory signals that dampen anti-tumor immune responses.

GSLs represent a largely untapped reservoir of potential targets for cancer immunotherapy. Despite their abundance and specificity in tumor membranes, they remain underutilized in clinical oncology. Designing high-affinity ligands or antibodies against GSLs has proven challenging due to their conformational flexibility and the structural complexity of glycan epitopes. One exception is dinutuximab, an anti-GD2 antibody approved for the treatment of high-risk neuroblastoma, with side effects such as severe pain and neuropathy. To circumvent the challenges associated with antibody development against GSLs, researchers have started to use STxB, a natural GSL-binding protein with high affinity for Gb3, overexpressed in several human tumors (e.g., breast, colorectal, gastric, pancreatic cancers, and lymphomas) [222–228]. Preclinical studies demonstrated that STxB selectively accumulates in Gb3-expressing tumors and maintains targeting efficacy after repeated administrations, due to its low immunogenicity [229, 230]. Synthetic approaches have been employed to conjugate STxB to various cytotoxic payloads, including: SN38 (topoisomerase I inhibitor) [224, 231], RO5-4864 (a pro-apoptotic benzodiazepine) [232], doxorubicin (intercalating agent) [233], MMAF (a potent tubulin polymerization inhibitor) [233]. These STxB–drug conjugates exhibited high specificity and nanomolar potency in vitro. However, in vivo efficacy was limited, likely due to off-target accumulation in kidneys, where Gb3 is also highly expressed. To address this, a proprietary platform has been developed to evolve STxB variants, through phage display selection, capable of selectively recognizing tumor-specific GSLs or GSLs combinations, potentially unlocking more selective therapeutic options [234].

Beyond targeted cytotoxicity, STxB has shown significant promise as a vaccine delivery vector. Its receptor Gb3 is naturally expressed on dendritic cells in different species, including human, enabling targeted antigen delivery for immune modulation [235–237]. When chemically coupled to tumor or virus-derived epitopes, STxB-antigen conjugates have demonstrated protective effects in models of viral infection and tumor progression [237–248]. Key immunological features of STxB-based vaccines include synergy with immune checkpoint inhibitors (e.g., anti-PD-L1) [243], and mucosal immunogenicity, with local IgA and tissue-resident CD8⁺ memory T cells—a critical population for tumor control [249]. Recent advances in chemical synthesis and in vitro folding of STxB further broaden the scope for customizing this platform, enhancing scalability and industrial development for future clinical translation [250]. Of interest, latest FDA regulations now classify synthetic peptides longer than 40 amino acids as biologicals. This change simplifies the regulatory process for projects developing synthetic proteins like STxB for clinical use.

In lysosomal storage disorders, which are mostly due to accumulation of GSLs, several therapies target GSL metabolism: enzyme replacement therapies such as imiglucerase (Gaucher) [251] and agalsidase (Fabry) [252], as well as substrate reduction therapies including miglustat [253] and eliglustat [254]. New oral GSL synthase inhibitors like lucerastat (phase 3 clinical trial NCT03425539) [255] and venglustat (Phase 3 clinical trial NCT05222906) [256] are in late-stage development, underscoring the translational potential of GSL biology.

## Outstanding questions and future directions

Despite remarkable progress, many questions remain on the biology of GSLs. The structural understanding of mechanisms by which GSLs exert allosteric control over membrane protein functions is still in its infancy. The phenomenal progress in cryogenic electron microscopy may offer new solutions, even if the flexibility of glycans remains a major challenge to structural explorations.

In the context of their contribution to building endocytic sites, GSLs offer a wealth of discovery opportunities. It remains unresolved through what mechanism(s) the interaction with GSLs becomes efficient only after galectin oligomerisation. Furthermore, it is still unclear how galectin oligomers that are nucleated on glycosylated cargo proteins reach onto the membrane to then reorganize GSLs for membrane curvature generation. Finally, the full scope of galectins, cargoes, and tissue contexts of GL-Lect driven endocytosis needs to be determined. A combination of structural and physiological approaches will be needed to go after these questions. Integrating lipidomics, glycomics, and imaging holds the promise of decoding broader principles that link structural diversity to cellular function and organismal physiology.

On the translational side, it is essential to determine how alterations in GSL metabolism contribute to diseases such as cancer, neurodegeneration, and immune dysfunction, and the extent to which GSLs can be exploited as therapeutic targets or delivery tools. Strategies under exploration include developing highly specific GSLs targeting ligands, leveraging GSL-rich membrane domains for targeted delivery and modulating GSL biosynthesis.

While antibody-based therapies have transformed oncology, designing antibodies against GSLs is challenging due to their structural flexibility and complex glycan epitopes. Emerging approaches aim to harness natural or engineered GSL-binding proteins as drug carriers or vaccine platforms. This can be achieved by evolving STxB or CTxB variants, capable of selectively recognizing tumor-specific GSLs or GSLs combinations, potentially unlocking more selective therapeutic options. Coupling of antigens to GSL-targeting scaffolds has shown promise in enhancing immune responses, including synergy with checkpoint inhibitors and mucosal immunity, which are critical features for durable protection against viral infections and tumors. In parallel, therapies for lysosomal storage disorders have advanced through enzyme replacement and substrate reduction. Notably, these approaches will require precise mechanistic insights to ensure specificity and minimize off-target effects.

## Conclusion

As the field of GSLs biology continues to advance, addressing the above key questions will enhance our understanding of molecular mechanisms in the context of health and disease. Addressing these gaps will require integrated approaches. Notably, recent advances in chemical biology and imaging are beginning to reveal mechanisms that link the structural diversity of GSLs to their cellular functions. At the same time, translational opportunities have started to expand from targeted drug delivery and immunotherapy to therapies for lysosomal storage disorders. It can thereby be expected that unlocking the full potential of GSL biology will provide not only deeper insight into membrane biology, but may also favor the emergence of innovative strategies for precision medicine.

## Data Availability

No data and material was used for the research described in this article.

## Declarations

Ethics approval and consent to participate.

Not Applicable.
